# Prognostic significance of cyclin D3 expression in malignancy patients: a meta-analysis

**DOI:** 10.1186/s12935-019-0865-3

**Published:** 2019-06-10

**Authors:** Bo Wang, Zijian Wang, Lizhi Han, Song Gong, Yanxue Wang, Zhiwen He, Yong Feng, Zhaohui Yang

**Affiliations:** 1 Department of Rehabilitation, Union Hospital, Tongji Medical College, Huazhong University of Science and Technology, Wuhan, 430022 China; 2 Department of Orthopedics, Xiangyang Central Hospital Affiliated Hubei University of Arts and Science, Xiangyang, 441021 China; 3 Department of Orthopaedics, Union Hospital, Tongji Medical College, Huazhong University of Science and Technology, Wuhan, 430022 China

**Keywords:** Cyclin D3, Prognosis, Malignancy, Meta-analysis

## Abstract

**Background:**

As a pivotal regulator, cyclin D3 gives play to a crucial value in conversion from the G1 stage to the S stage of cell cycle, which is implicated in tumor progression, especially proliferation and migration. Recent literatures have reported that cyclin D3 could predict survival time of malignancy patients. But, its prognostic role of cyclin D3 in neoplasms remains controversial.

**Methods:**

Databases involving EMBASE, PubMed and Web of Science were carefully searched, and literatures investigating the prognostic effect of aberrantly expressing cyclin D3 among human cancers were collected for further analysis. We used both hazards ratios and its corresponding 95% confidence intervals to evaluate the connection among the survival rate of malignancy patients and the expression of cyclin D3.

**Results:**

There were 13 eligible researches involving 16 cohorts and 2395 participants which were included in this study. The outcomes suggested that highly expressing cyclin D3 was significantly correlated with worse clinical prognosis of overall survival (HR 1.88; 95% CI 1.31–2.69) and disease specific survival (HR 2.68; 95% CI 1.35–5.31). But there existed no significant connection between the elevated expression of cyclin D3 with disease free survival (HR 2.65; 95% CI 0.83–8.46), recurrence-free survival (HR 2.86; 95% CI 0.82–9.96) and progression-free survival (HR 5.24; 95% CI 0.46–60.25) of diffident kinds of malignancy patients. Moreover, we discovered that elevated cyclin D3 expression was significantly connected with decreased overall survival in lymphoma (HR 3.72; 95% CI 2.18–6.36) while no significant relevance between highly expressing cyclin D3 and the overall survival in breast cancer was obtained (HR 2.12; 95% CI 0.76–5.91).

**Conclusions:**

This meta-analysis demonstrated that highly expressing cyclin D3 might be an unfavorable prognostic biomarker for various malignancy patients, which can make great contributions to the clinical diagnosis and treatment.

## Background

With the medical technology rapidly developing and the global environment seriously contaminated, there are more and more people diagnosed with diverse cancers, like mammary carcinoma, pulmonary neoplasm and so forth, which burdens the global medical system, and the cost about it accounts for the majority of global medical insurance [1]. Many doctors have taken positive measures, surgery or radiochemotherapy, to curb the current growing number of tumor patients. Nowadays, the scientist and pharmacologist have made a remarkable breakthrough in developing a new therapy method called targeted-therapy, which is of epoch-making significance for curing tumor and drug-resistant patients. However, owing to lack of related prognostic proofs, the targeted proteins about oncogenesis are not well characterized [2]. Thus, it is essential to discover a lot of new targets for human cancers.

Cyclin D3 belongs to the D-Cyclin proteins family, which can act as a crucial regulator to the differentiation and proliferation of tumor cells [3, 4]. In the G1 stage, Cyclin D proteins were found to be high-expressed and associated with their kinase partners CDK4 and CDK6. Also, it is possible for them to regulate the G1 restriction point of cell cycle via phosphorylating the retinoblastoma protein [5, 6]. Additionally, regulation of G1/S transition, a common biochemical pathway, is a key target of tumorigenesis because cells which have developed to S stage can be devoted to cell division. And D-Cyclin proteins consisting of cyclin D1, D2, and D3 can be up-regulated via signals of growth-promotion, which can associate mitogenic signals with cell cycle machinery. All of them are indispensable to the development of G1 and can limit the G1/S transition rates [7]. Therefore, these D-Cyclin proteins could be concern with tumorigenesis.

According to gene sequencing, bioinformatics and experiments with transgenic mice or nude mouse tumorigenicity, both cyclin D1 and D2 have been proved to be part of proto-oncogenes. In term of cyclin D1, many studies have demonstrated that this protein is associated with a poor prognosis in different kinds of cancers [8–10]. Recently, five systematic reviews and meta-analysis have reported that cyclin D1 could be a prognostic biomarker for various carcinoma [11–15]. As for cyclin D2, some studies also reported that it might be related with worse prognosis in some tumors [16–18]. And a genome-wide meta-analysis has identified cyclin D2 as genetic susceptibility loci for colorectal tumors [19]. The above all have proved that the prognosis role of both cyclin D1 and D2 are well clarified. However, the effects of cyclin D3 has not been explained clearly.

6p21 chromosome region is able to encode cyclin D3 and the corresponding expression protein is mainly located in the nucleus [20, 21]. There is a large body of evidence that indicate that aberrantly expressing cyclin D3 have been found in different kinds of neoplasms [22–26] and even linked to many malignant phenotypes [27–32]. In addition, Chen et al. [33] had clarified that cyclin D3 is likely to become a critical molecular target for antitumor chemotherapeutic purpose in mammary carcinoma patients. Furthermore, Jeffrey et al. [34] likewise had revealed that high expressing cyclin D3 is relevant with erlotinib resistance in respiratory and digestive tumors. Hence, it is of great importance for us to study whether high-expression cyclin D3 is correlative with poor prognosis and cyclin D3 is a key point of chemotherapy for cancer. However, its potential role in prognostication is restrictedly reported, which obviously limits its pharmaceutical prospects [35].

Thence, a quantitative meta-analysis was carried out aiming to assess the prognosis and predictive significance of the expressions of cyclin D3 in human malignancy and offer more theories for clinical applications.

## Materials and methods

### Study strategy

All procedures mentioned below were conducted based on a standard guideline for meta-analysis involving cancer biomarker prognosis trials [36, 37]. Two researchers performed each step individually, while any disagreement was solved by group conference. Databases of EMBASE, PubMed and Web of Science were independently searched by two researchers to acquire the related literatures involving the prognosis significance of cyclin D3 expression among malignancy sick personnel. For the sake of enhancing sensitivity of our search, not only free-text words but also MeSH terminology were utilized in current meta-analysis. The search strategy was: “CCND3 or cyclin D3” AND “neoplasms or neoplasias or tumors or cancers or carcinoma or malignancies or malignant neoplasms” AND “prognoses or prognostic factors or prognostic or prognosis or survival or outcome”. And related references of searching concerned literatures were also screened to identify potentially eligible literatures.

### Inclusion and exclusion criteria

Researches that complied with the undermentioned criteria were eventually enrolled: (1) Patients were pathologically diagnosed with any type of malignancy. (2) The expression levels of cyclin D3 were identified in tissues samples. (3) Patients were classified into negative and positive expression or low and high expression group in line with the cyclin D3 of expression levels, the connection between expressing level of cyclin D3 and survival results was examined. (4) Hazard ratios (HR) and their 95% confidence intervals (CI) for survival times were computed by included articles which can provide enough data or survival curves. (5) Officially published and English-written literatures until July 2018. The eliminated criteria as follows: 1. Duplicated articles; 2. Reviews, laboratory articles, case-reports and conference abstracts; 3. Insufficient data about survival analysis.

### Data extraction

Two investigators extracted related data respectively and came to an agreement on the following items. Original data of elementary demographic characteristics (year of publication; authors of article; region; the category of carcinoma; detection method; cyclin D3 level; sample size; age; follow-up duration; Newcastle–Ottawa Scale score (NOS); cut-off value and endpoints) were exhaustively extracted from included literatures involving Kaplan–Meier curves, test words and tables. In term of endpoints, overall survival (OS), progression-free survival (PFS), recurrence-free survival (RFS), disease free survival (DFS) and disease specific survival (DSS) were considered as terminal events. For the purpose of evaluating the effect of the expression level of cyclin D3 for cancer patients in prognosis, HR was adopted via abiding by a methodology recommended previously [38]. Furthermore, original data was also obtained by contacting the authors of the included literatures.

### Methodological assessment

Two investigators individually assessed qualities of all enrolled researches by utilizing the Newcastle–Ottawa Scale. The critical scale is divided into three categories consisting of outcomes, selection of subjects and comparability of trial groups to assess a study. We regarded the included studies with at least six score as high-quality in methodology. And if the scores were less than 6, those articles were considered as low-quality studies.

### Statistical analysis

Our quantitative calculation was conducted based on Stata Software 14.0. We applied pooled HRs (high/low) along with its related 95% CIs to evaluate the association between the prognostic value and the expression levels of cyclin D3 in different malignancies. By utilizing Cochran’s Q and I^2^ statistics, the heterogeneity of enrolled literatures can be evaluated precisely. Additionally, we regarded an I^2^ value larger than 50% or a p value no more than 0.10 as statistically significance. An insignificant heterogeneity (p > 0.10, I^2^ < 50%) was changed via the fixed-effects model for analysis. On the contrary, we would select the random-effects model. In order to explore the source of heterogeneity, we also performed subgroup analysis and meta-regression. Furthermore, sensitivity analysis was implemented to confirm the steadiness of collected results. Finally, we assessed publication bias by means of utilizing both Begg’s and Egger’s test. What’s more, if the p value is no more than 0.05, the results above all can be regarded as statistical significance.

## Results

### Characteristics of studies

**Fig. 1:**
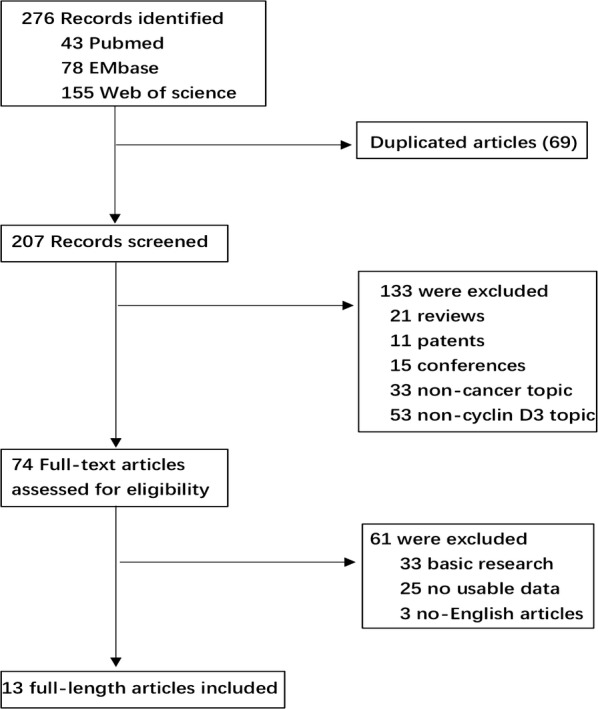
The flow diagram indicated the process of study selection

Eventually, we selected 13 studies [39–51] from the initial retrieved 276 literatures consisting of 16 cohorts. The specific selection flow chart of each steps was illustrated in Fig. 1. In addition, the entire sample size of included studies ranged from 66 to 391, which added up to 2395 participants. What’s more, the range of follow-up duration in all enrolled cohorts was from 30 to 168 months. In term of various types carcinomas, there were three different kinds of tumors that were the most including breast neoplasms (n = 2), urinary bladder neoplasms (n = 2) and lymphoma (n = 3). Among these studies, OS (n = 11), DFS (n = 2), RFS (n = 2), FRS (n = 2) and DSS (n = 1) were regarded as survival outcome. When it comes to the detection methods for analyzing the expression of cyclin D3, immunohistochemistry (IHC) was the most frequent method, following by polymerase chain reaction (PCR) and the rarest was western blot (WB). Finally, the values of cut-off were varied from each study owing to the various definitions for cut-off. Further details about baseline features were recorded in Table 1.

**Table 1:** Characteristics of studies included in the meta-analysis

Study	Region	Tumor type	Detection method and main results	Sample size (high/low)	Follow-up (month)	Endpoints	Cut-off value	Mean age (year)	NOS score	Method
Filipits et al. [39]	Austria	Diffuse large B-cell lymphoma	IHC (cyclin D3, 30/100)	31/50	30	OS	High: more than 50% cyclin D3-positive lymphoma cells	NA	7	1
Chi et al. [40]	China	Breast cancer	IHC (cyclin D3, NA)	170/73	60	OS, DFS	High: moderate/strong nucleus staining	NA	6	1
Huang et al. [41]	China	Hepatocellular carcinoma	PCR (cyclin D3, NA)	88/92	105.6	OS	Median	NA	6	2
Lopez-Beltran [42]	Spain	Bladder cancer	IHC (cyclin D3, 1000)	9/150	74.7 ± 28.0	PFS	High: more than 25% cyclin D3-positive cancer cell	61	6	2
Pruneri et al. [43]	Italy	Laryngeal squamous cell carcinoma	IHC (cyclin D3, 300)	88/135	52	OS, PFS	High: more than 10% cyclin D3-positive cancer cell	61.5	8	1
Keyomarsi et al. [44]	USA	Breast cancer	WB (cyclin D3, NA)	115/272	76.8	OS, DFS	High: higher than the level in normal-cell controls, indicated by a score of more than 2	76.8	8	2
Levidou et al. [45]	Greece	Ovarian adenocarcinomas	IHC (cyclin D3, 1000)	78/14	41.5	OS	Percentage of neoplastic cells with clear nuclear immunoreactivity out of the total number of neoplastic cells counted	57	7	2

Study	Region	Tumor type	Detection method (number of analyzed cells)	Sample size (high/low)	Follow-up (month)	Endpoints	Cut-off value	Mean age (year)	NOS score	Method
Moller et al. [46]	USA	Non-Hodgkin lymphoma	IHC (cyclin D3, 1000)	43/155	60	OS, RFS	High: more than 5% cyclin D3-positive cancer cell	NA	7	2
Lopez-Beltran [47]	Spain	Bladder cancer	IHC (cyclin D3, 1000)	21/138	74.8 ± 28.1	OS	High: more than 13% cyclin D3-positive cancer cell	61	6	2
Florenes et al. [48]	Norway	Malignant melanoma	IHC (cyclin D3, NA)	22/85	100	OS, RFS	High: more than 5% cyclin D3-positive cancer cell	NA	5	1
Chen et al. [49]	China	Central nervous system lymphoma	IHC (cyclin D3, NA)	35/31	30	OS	High: more than 20% cyclin D3-positive cancer cell	52.8	7	1
Sterlacci et al. [50]	Austria	Non-small cell lung cancer	IHC (cyclin D3, NA)	167/224	168	OS	High: any staining of tumor cell membranes above background level	62	7	2
Hedberg et al. [51]	Sweden	Renal cell carcinoma	IHC (cyclin D3, 300)/WB	24/164	88.5	DSS	High: tumors with > 5% positive cells	65.12	6	2

Method: 1 denoted as obtaining HRs directly from publications; 2 denoted as HRs calculated from the total number of events, corresponding p value and data from Kaplan–Meier curves

*IHC* immunohistochemistry, *PCR* polymerase chain reaction, *WB* western blot, *OS* overall survival, *DFS* disease free survival, *RFS* recurrence-free survival, *PFS* progression-free survival, *DSS* disease specific survival, *NOS* Newcastle–Ottawa Scale, *NA* not available

### Relationship between cyclin D3 expression level with OS of malignancy patients

**Fig. 2:**
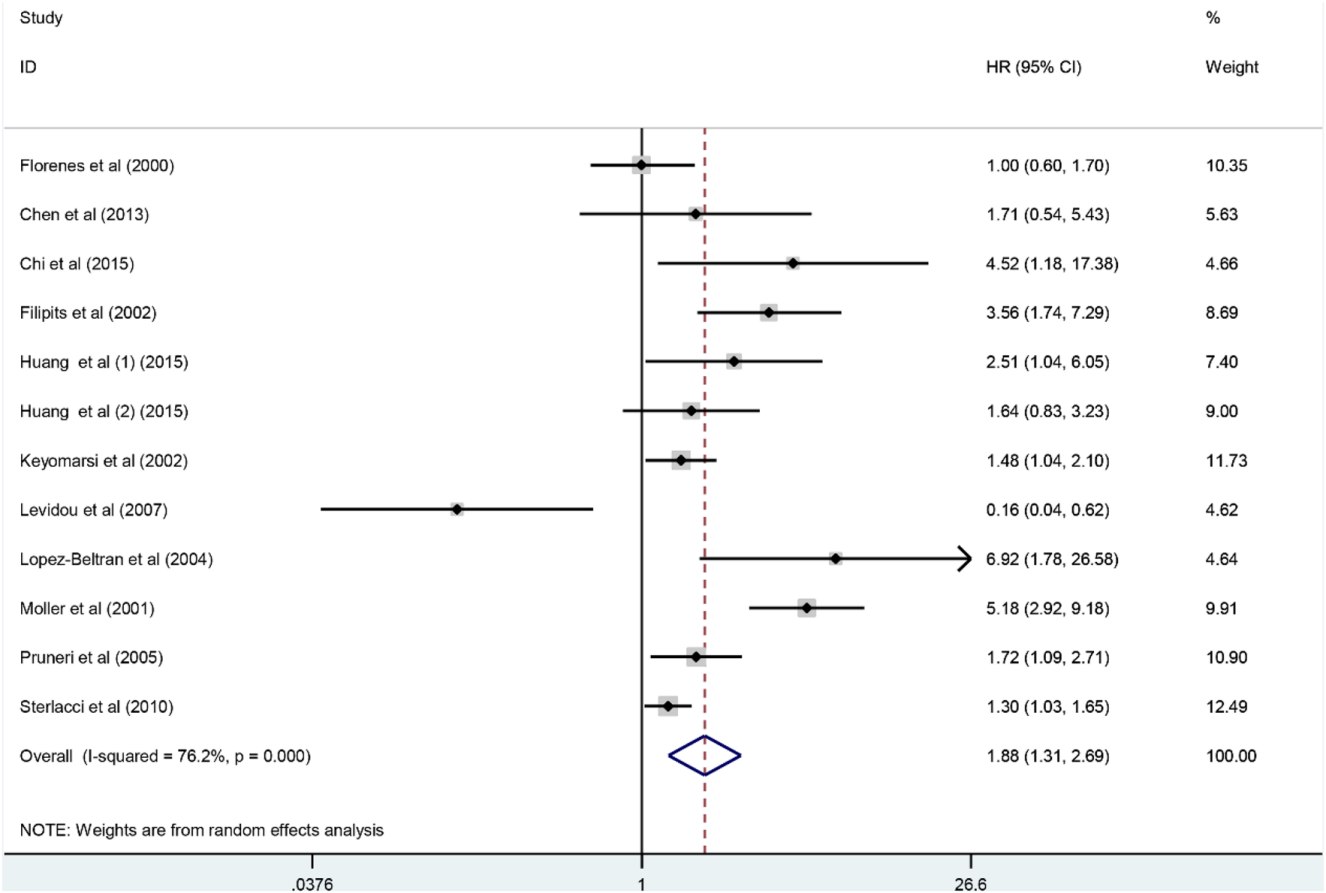
Meta-analysis of the pooled HR of OS for malignancy patients

**Fig. 3:**
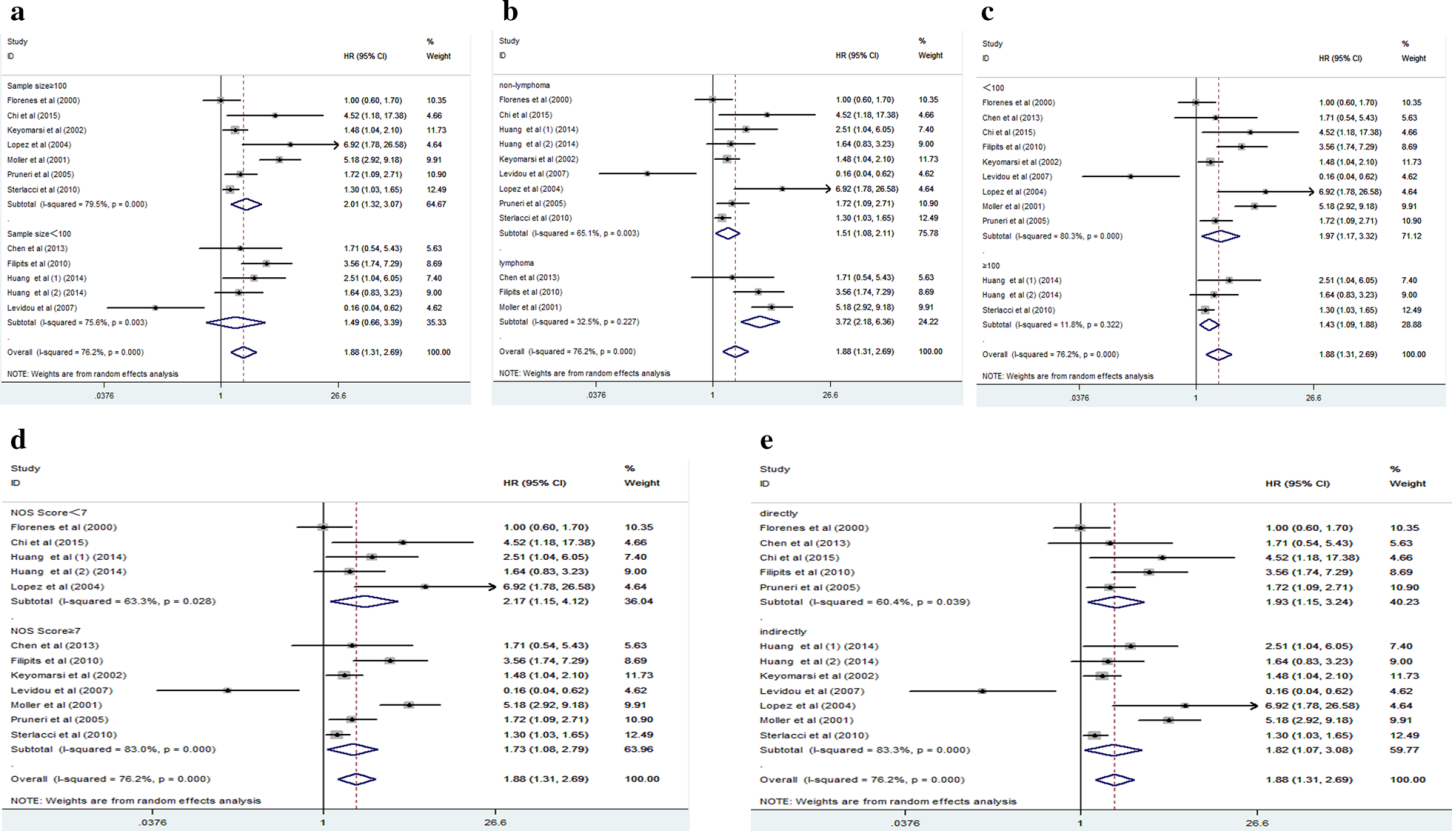
Results of subgroup analysis of pooled HR of OS for malignancy patients. **a** Subgroup analysis stratified by sample size. **b** Subgroup analysis stratified by type of cancer. **c** Subgroup analysis stratified by follow-up time. **d** Subgroup analysis stratified by NOS score. **e** Subgroup analysis stratified by source of HR

There were eleven studies exploring the association between aberrantly expressing cyclin D3 with OS in this meta-analysis. At the same time, we applied random-effect model to reckon the pooled HR. And result demonstrated that higher expression level of cyclin D3 was significant correlated to reduction of OS among malignancy patients (HR 1.88; 95% CI 1.31–2.69, p = 0.001) (Fig. 2). Owing to obvious heterogeneity from all included studies (I^2^ = 76.2%, p < 0.001), subgroup analysis was further performed by factors of type of malignancy (lymphoma or non-lymphoma), sample size (more than 100 or less than 100), access of HR (directly or indirectly), follow-up duration (over 100 or less than 100 months) and the quality of enrolled literatures (NOS scores < 7 or ≥ 7) to investigate sources of heterogeneity (Fig. 3a–f). Consequently, our outcomes of subgroup analysis demonstrated that relationship between cyclin D3 redundancy and worse OS of malignancy patients remained notable except for the subgroup of less than 100 participants (p = 0.338) (Table 2). To further explore the source of heterogeneity, we performed meta-regression by the covariates including above factors. But, the results of meta-regression did not reveal p values no more than 0.05 in above covariates, which indicated that all above factors were not the sources of heterogeneity (Table 2).

**Table 2:** Subgroup analysis of pooled HRs for OS in cancer patients with abnormal expression level of cyclin D3

Subgroup analysis	No. of cohorts	Pooled OR Random	Meta regression (p value)	Heterogeneity	
				I^2^ (%)	p value
Sample size			0.517		
≥ 100	7	2.01 [1.32–3.07]	–	79.5	0.000
< 100	5	1.49 [0.66–3.39]	–	75.6	0.003
Source of HR			0.833		
Directly	5	1.93 [1.15–3.24]	–	60.4	0.039
Indirectly	7	1.82 [1.07–3.08]	–	83.3	0.000
NOS scores			0.571		
≥ 7	7	1.73 [1.08–2.79]	–	83.0	0.000
< 7	5	2.17 [1.15–4.12]	–	63.3	0.028
Follow-up time			0.800		
< 100	9	1.97 [1.17–3.32]	–	80.3	0.000
≥ 100	3	1.43 [1.09–1.88]	–	11.8	0.322
Type of cancer			0.077		
Lymphoma	3	3.72 [2.18–6.36]	–	32.5	0.227
Non-lymphoma	9	1.51 [1.08–2.11]	–	65.1	0.003

### Relationship between the expression of cyclin D3 with OS of certain type of malignancy

**Fig. 4:**
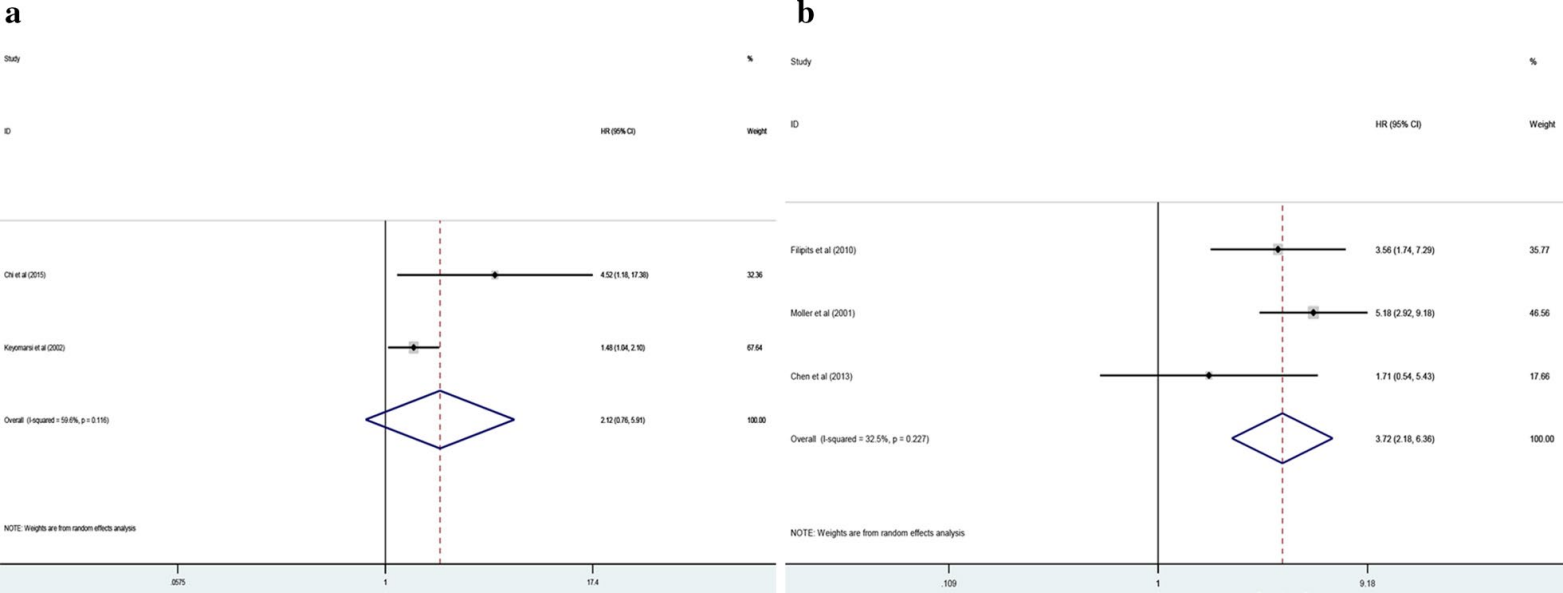
Meta-analysis of the pooled HR of OS for breast cancer (**a**) and lymphoma (**b**)

Additionally, the prognosis role of the expression levels of cyclin D3 in two kinds of cancers was assessed systemically. Our outcomes suggested that elevated cyclin D3 level implicated an unfavorable OS in lymphoma (HR 3.72; 95% CI 2.18–6.36, p < 0.000) (Fig. 4a). Nevertheless, regarding breast neoplasms, our results revealed that no significant relationship between the cyclin D3 redundancy with OS was obtained (HR 2.12; 95% CI 0.76–5.91, p = 0.149) (Fig. 4b).

### Relationship between cyclin D3 expression level with DFS, RFS, PFS and DSS of malignancy patients

**Fig. 5:**
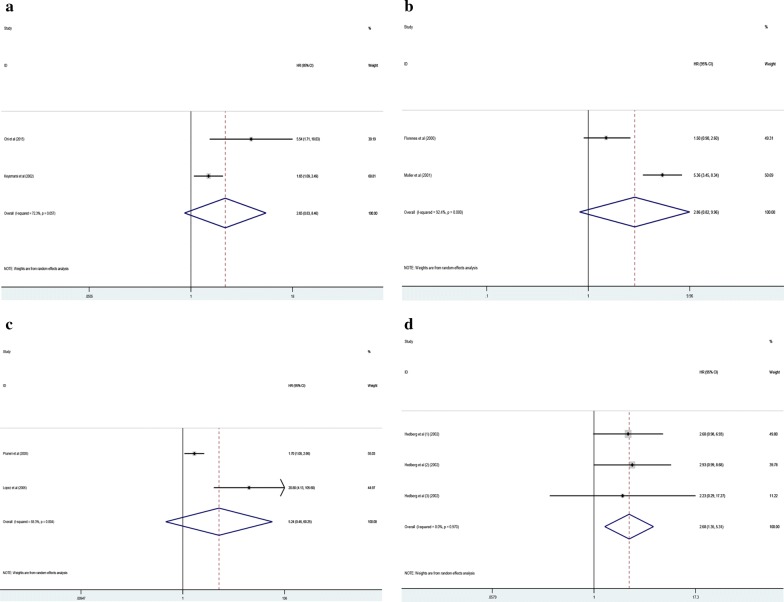
Meta-analysis of the pooled HR of DFS (**a**), RFS (**b**), PFS (**c**) and DSS (**d**)

Among the included studies, two researches estimated the relevance between cyclin D3 expression level with DFS, RFS and PFS, respectively. And only one study including three cohorts evaluated the connection between the expression level of cyclin D3 with DSS. Among this meta-analysis, we showed that cyclin D3 increasingly expressing had significantly worse outcome in worse DSS (HR 2.68; 95% CI 1.35–5.31, p = 0.005) (Fig. 5d). However, no matter how high or low expression level of cyclin D3, there existed no differentiation in DFS (HR 2.65; 95% CI 0.83–8.46, p = 0.099) (Fig. 5a), RFS (HR 2.86; 95% CI 0.82–9.96, p = 0.099) (Fig. 5b) and PFS (HR 5.24; 95% CI 0.46–60.25, p = 0.184) (Fig. 5c). Moreover, we did not carry out the subgroup analysis thanks to the finite numbers of included trials.

### Sensitivity analysis

**Fig. 6:**
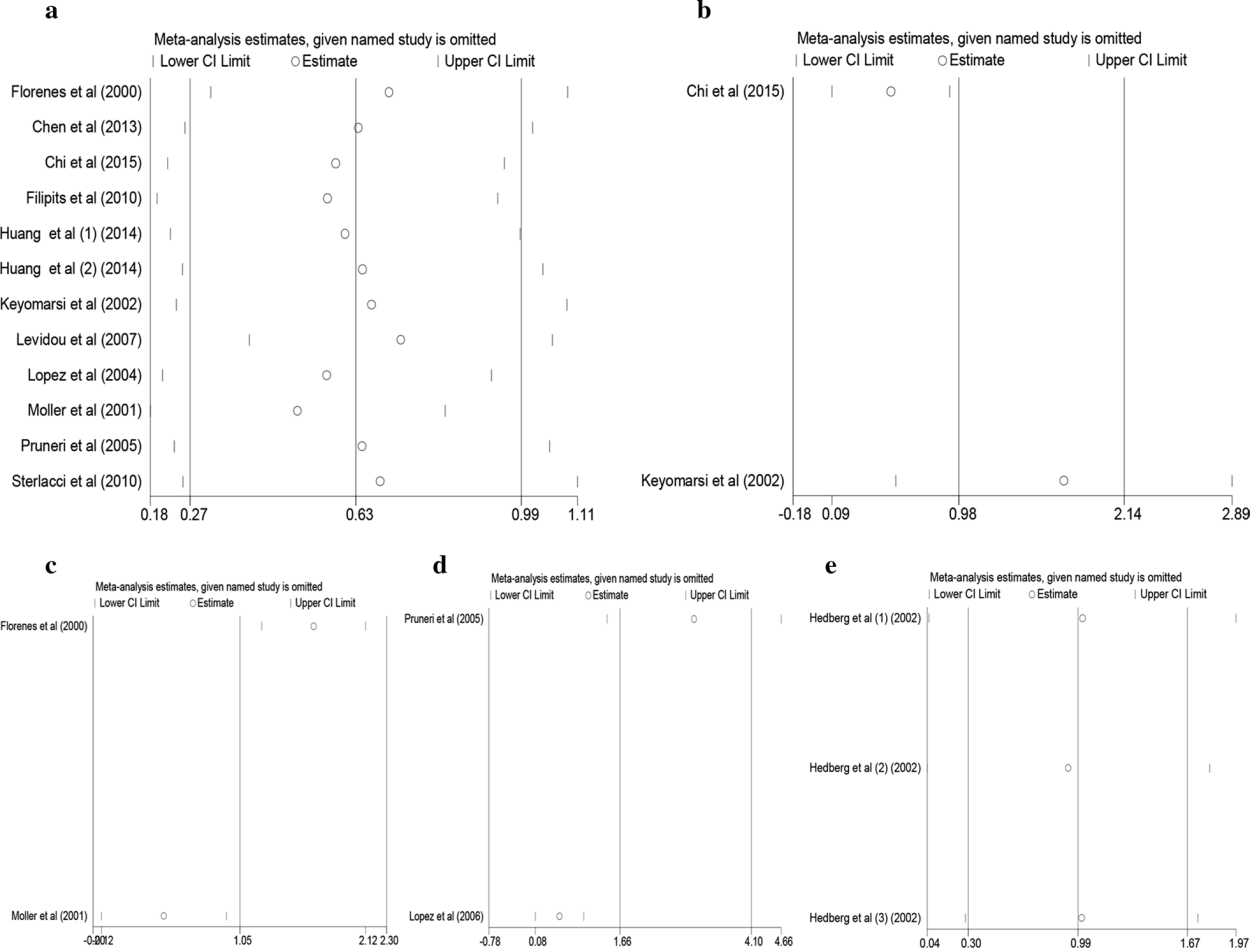
Sensitivity analysis plot of pooled HR of OS (**a**), DFS (**b**), RFS (**c**), PFS (**d**) and DSS (**e**) for malignancy patients with abnormally expressed level of cyclin D3

In order to assess the impacts of single study on the total outcomes, sensitivity analysis was conducted. With respect to OS, our result of sensitivity analysis revealed that the outcomes originating from Moller et al. and Levidou et al. influenced consequences remarkably, demonstrating that the critical source of heterogeneity was likely to come from the above studies. The list of pooled HRs and 95% CIs after excluding single study one by one indicated robustness of our results (Fig. 6a). Furthermore, with regard to DFS (Fig. 6b), RFS (Fig. 6c), PFS (Fig. 6d) and DSS (Fig. 6e) our sensitivity analysis identified that all selected studies influenced outcomes greatly, which suggested that the outcomes of DFS, RFS, PFS and DSS were not stable because of the limited number of studies included in each analysis. Thus, more and more related studies were needed to explore the effects of cyclin D3 on DFS, RFS, PFS and DSS in human malignancy.

### Publication bias

**Fig. 7:**
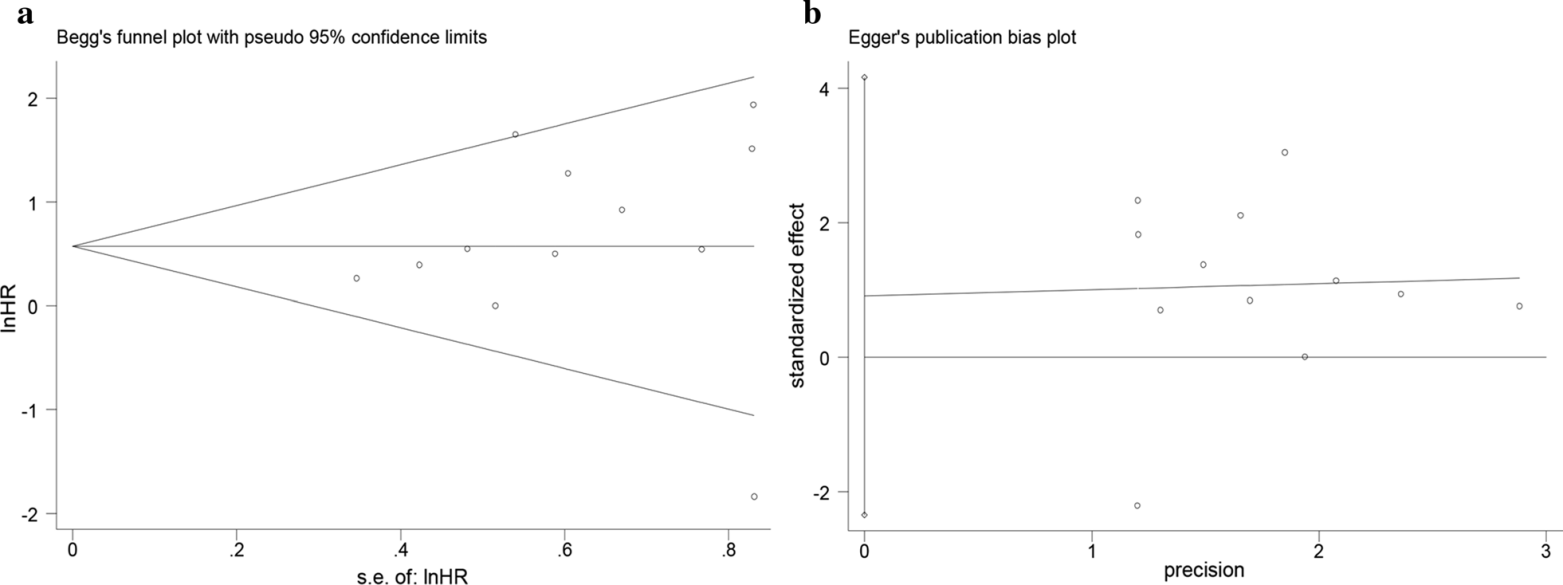
Begg’s test (**a**) and Egger’s test (**b**) for publication bias

By using Begg’s test and Egger’s test, we systemically assessed publication bias of all above included studies. The result of Begg’s test (p = 0.273) (Fig. 7a) and Egger’s test (p = 0.547) (Fig. 7b) about OS revealed that there existed no significant publication bias among enrolled documents. In terms of DFS, RFS, PFS and DSS, we didn’t perform the publication bias just because of the small amount of selected literatures, no more than 10 in each analysis.

## Discussion

Accordingly, our quantitative outcomes illustrated that elevated cyclin D3 expression indicated unfavorable prognosis including both OS and DSS among diverse malignancy patients. What’s more, we assessed the prognosis role of cyclin D3 among two kinds of malignancies. We just discovered that high expressing cyclin D3 was related to decreased OS in lymphoma.

The above conclusions appear to be rational and understandable in line with the current agreement that as a chief cancer promoter, cyclin D3 can promote the abnormal growth and tumorigenesis of different kinds of tumors, such as diffuse large B cell lymphoma, which can serve as a promoter for regulating the progression of G1/S transition and the survival of cancer cells [52, 53]. What’s more, Choi et al. had demonstrated that besides the ingrained values of overexpressed cyclin D3 in tumor initiation, the presence of cyclins D3 is essential for tumor maintenance [54], which jointly contributes to the unfavorable prognosis in patients with elevated cyclin D3 expression levels. But one of studies that differ from the majority included cohorts suggested that high-expression cyclin D3 is related to better OS and DFS in nodular melanoma [48], while the conclusion was interestingly opponent in superficial melanoma. However, in this study, there was no statistically significant between the high expression levels of cyclin D3 and overall survival for nodular melanoma patients (p = 0.23). Thus, cyclin D3 is not likely to act as a prognosis factor for the nodular melanoma patients and other proteins or pathways might be at work to promote nodular melanoma and play a prognostic role.

On account of the significant heterogeneity among included studies, both subgroup analysis and meta-regression analysis were used to investigate origins of heterogeneity. As a consequence, our outcomes of subgroup analysis revealed that sample size (over 100 or less than 100) changed the significant prognostic value of cyclin D3 in OS (HR 2.01; 95% CI 1.32–3.07 vs HR 1.49; 95% CI 0.66–3.39). This suggested that the root of heterogeneity may be from the distinctness existing in each sample sizes. Nevertheless, our meta-regression analysis couldn’t acquire the origination of the significant heterogeneity in above all factors.

Furthermore, we also explored the association between the cyclin D3 expressing levels with the prognostic value among various cancers. But just on account of the restricted amounts of selected researches, we only evaluated the prognostic value of cyclin D3 in mammary tumor and lymphoma. And the results showed that higher cyclin D3 level implicated an unfavorable OS in lymphoma patients. However, we just found that there existed no difference for the elevated expression levels of cyclin D3 to forecast the OS of breast neoplasms. The reason may be that the included studied Chi et al. only evaluated the prognostic role of cyclin D3 in stages I-III of breast cancer. Similarly, breast cancer patients in stages I-II account for 80% of the total participants in the study of Keyomarsi et al. Based on the above, we speculate that the prognostic roles of cyclin D3 might be different in diverse cancer stage. Therefore, a growing number of larger-scale, multicenter studies including all stage patients are needed to verify our hypothesis.

With regard to DFS, RFS, PFS and DSS, these are all essential parameters reflecting the procession of malignancy. The outcomes of this meta-analysis revealed that higher cyclin D3 level implicated an unfavorable DSS in tumor patients. Nevertheless, no matter how high or low cyclin D3 expressed, there existed no difference in forecasting the DFS, RFS and PFS of tumor patients. What’s more, owing to the fact that only two researches were enrolled to appraise the connection among the cyclin D3 expressing levels and DFS, RFS and PFS respectively, more and more researches are essential to investigate the connection about cyclin D3 and the development of cancer.

Except for the encouraging results, there are several limitations among this quantitative meta-analysis. First and foremost, in spite of using both random-effects model and subgroup analysis, we are unable to remove the heterogeneity across researches leading to some bias of the results to some extent. Second, the cut-off value of cyclin D3 expressing levels was varied among our included researches, which could cause the bias of the results. Moreover, our summary analysis fully depends on the strength of including cohort above all, thereby selection bias might exist in our outcomes. Finally, some hazard ratios are not able to acquire from the included literatures directly. Therefore, the results might not be accurate enough by survival curves.

## Conclusions

In sum up, this meta-analysis suggested that higher expressing levels of cyclin D3 was correlative to worse prognosis of OS, DSS among different kinds of malignancy patients. Nevertheless, there existed no remarkable connection in both cyclin D3 expressing levels and DFS, RFS and PFS in our study. In brief, our current study is the earliest meta-analysis that systemically explores the incontrovertible evidence of the prognosis value of cyclin D3 in various malignancy patients. More and more related researches are needed to explore the value of cyclin D3 in different kinds of cancers.

## Data Availability

The datasets during and/or analysis during the current study available from the corresponding author on reasonable request.

## Abbreviations

OS: overall survival
PFS: progression-free survival
RFS: recurrence-free survival
DFS: disease-free survival
DSS: disease specific survival
HR: hazard ratios
CI: confidence intervals
IHC: immunohistochemistry
WB: western blot
PCR: polymerase chain reaction
NOS: Newcastle–Ottawa Scale
